# Neoadjuvant Therapy for Locally Advanced Gastric/Gastroesophageal Junction Adenocarcinoma: Current Status, Challenges, and Future Perspectives

**DOI:** 10.1002/cam4.71753

**Published:** 2026-03-24

**Authors:** Jiaqi Zhou, Chengwang Guo, Mingze Zhang, Xi Guo, Jihong Wang, Xiaojun Liu, Peng Nie, Yuntao Ma

**Affiliations:** ^1^ The First Clinical School of Gansu University of Chinese Medicine Lanzhou Gansu China; ^2^ Department of General Surgery Mianyang 404 Hospital Mianyang China; ^3^ Department of Gastric Tumor Surgery Gansu Wuwei Tumor Hospital Wuwei Gansu China

**Keywords:** chemotherapy, gastric cancer, immunotherapy, neoadjuvant therapy, targeted therapy

## Abstract

Gastric cancer remains a leading cause of cancer‐related mortality worldwide, with locally advanced gastric/gastroesophageal junction adenocarcinoma posing significant therapeutic challenges. Neoadjuvant therapy has emerged as a critical strategy to improve surgical outcomes and long‐term survival by reducing tumor burden and eradicating micrometastases. This systematic review integrates evidence from more than 100 clinical trials and 20 meta‐analyses published in recent years. It focuses on new advances in neoadjuvant therapies for gastric cancer, including chemotherapy, radiotherapy, immunotherapy, and targeted therapies, while highlighting emerging combination strategies and unresolved clinical dilemmas.

## 1 Introduction

Gastric cancer (GC) is a prevalent malignant tumor of the digestive system. According to 2022 data from the Global Cancer Research Institute (GLOBOCAN), GC is the sixth most common cancer and the third leading cause of cancer‐related deaths worldwide [1, 2]. In recent years, the incidence of GC in China has been gradually declining [3]. However, with the aging population, the number of cases and deaths from GC remains high, posing a significant burden on the healthcare system [4]. In China, the early diagnosis rate of GC is low, and the characteristics of locally advanced tumors – heavy burden, strong heterogeneity, and poor prognosis among newly diagnosed GC patients – make diagnosis and treatment particularly challenging [5].

Traditional treatment for GC primarily relies on surgery, supplemented by adjuvant chemotherapy or radiotherapy. However, due to the complexity of locally advanced G/GEJ adenocarcinoma, treatment plans must consider a combination of surgery, chemotherapy, radiotherapy, immunotherapy, and targeted therapy [6, 7]. Neoadjuvant therapy has demonstrated significant advantages in improving the surgical resection rate, reducing the risk of recurrence, eradicating potential micrometastases, and prolonging patient survival [8, 9, 10]. With the continuous development of chemotherapy drugs, radiotherapy techniques, immunotherapy, and targeted therapy, the efficacy of NAT has been further improved. Nonetheless, challenges such as increasing individual differences in treatment effects and drug resistance persist [11]. This article reviews the current research status, issues, and challenges of NAT for G/GEJ adenocarcinoma.

## 2 The Current Status of Neoadjuvant Therapy for Gastric Cancer

### 2.1 Neoadjuvant Chemotherapy

#### 2.1.1 Research Status

Neoadjuvant chemotherapy (NAC), as a standard therapeutic regimen for locally advanced G/GEJ adenocarcinoma, has been demonstrated to significantly improve surgical resection rates and survival outcomes in clinical practice [12]. The successful experience of the MAGIC trial in Europe in 2006 marked a significant achievement in NAC for locally advanced gastric cancer (LAGC). In this study, 250 patients and 253 patients were included in the NAC with ECF regimen combined with surgery group and the surgery alone group, respectively. The study showed that the incidence of postoperative complications was similar (46% and 45%, respectively), and the number of deaths within 30 days after surgery was also similar. The study demonstrated that the perioperative ECF regimen could reduce tumor staging, significantly improve progression‐free survival (PFS), and overall survival (OS) [13]. In the randomized phase II/III FLOT4‐AIO study conducted in 2019, 360 patients were assigned to the ECF/ECX group, and 356 patients were assigned to the FLOT group. Compared with the ECF/ECX group, the OS of the FLOT group was improved (hazard ratio [HR] 0.77; 95% confidence interval [CI; 0.63–0.94]; median OS, 50 months [38.33 to not reached] vs. 35 months [27.35–46.26]). The number of patients with related serious adverse events and toxic deaths was similar between the two groups; the results confirmed that the FLOT regimen significantly improved OS compared with the ECF or ECX regimen [14]. Based on the FLOT4‐AIO study, the European Society for Medical Oncology (ESMO) and the National Comprehensive Cancer Network (NCCN) have both recommended the FLOT regimen as the preferred perioperative treatment for LAGC. Furthermore, a systematic review integrated with a Bayesian network meta‐analysis encompassing 12 randomized controlled trials (RCTs) and 3846 eligible participants demonstrated that the FLOT regimen significantly enhances multiple clinically relevant endpoints, including tumor resectability, disease‐free survival (DFS), and treatment safety profiles [15, 16]. An EORTC study conducted in Germany confirmed positive prognostic outcomes of NAT and demonstrated that the combination of cisplatin, folinic acid, and 5‐FU has lower toxicity compared to etoposide, doxorubicin, and cisplatin (EAP), while still providing a similar chemotherapy response [17]. Among the 41 patients enrolled in this study, 36 underwent surgery, with an R0 resection rate of 73.2% (*n* = 26). After a median follow‐up of 18.3 months, 23 of the R0‐resected patients remained alive, and 19 (73%) were free of recurrence. No severe complications or postoperative deaths were observed. Furthermore, a population study based on data from the Dutch Upper Gastrointestinal Cancer Audit (DUCA) showed a reduced utilization of NAC in GC patients aged 70 and above, possibly due to the higher risk of treatment cessation associated with older age [18]. Increasing the use of NAC is reliable in terms of surgical safety and may help achieve better pathological outcomes. However, since the fact that most of these clinical trials were conducted in Western countries, the perioperative regimens of ECF, CF, and FLOT were used less frequently in Asia [19].

The results of the PRODIGY III clinical trial in Korea showed that neoadjuvant DOC chemotherapy extended the PFS and OS of patients with LAGC in Asia [20]. According to the NEO‐CLASSIC study, which included Chinese patients, perioperative XELOX chemotherapy combined with radical gastrectomy for LAGC showed good efficacy, safety, and tolerability [21]. In this study, 54 patients were included in the outcome analysis, all of whom were treated with the XELOX regimen perioperatively. Among them, 45 patients (83.3%) achieved R0 resection, while 4 patients underwent R1 resection. No complete response (CR) was observed; 27 patients (50.0%) achieved partial response (PR), 22 patients (40.7%) maintained stable disease (SD), and 4 patients (7.4%) developed progressive disease (PD). Accordingly, the objective response rate (ORR) was 50.0%. The median follow‐up time was 52.97 months; 30 patients (55.6%) developed disease progression, yielding a median PFS of 20.10 months and a median OS of 30.77 months. The incidence of grade 3–4 adverse events (AEs) was 8.5%. The JCOG 0501 phase III study conducted by a Japanese research team confirmed that radical surgery after NAC is superior to adjuvant chemotherapy, and a post hoc analysis of this study indicated that NAC may reduce the risk of surgical infectious complications [22, 23]. Published in *The Lancet Oncology* in 2021, the phase III RESOLVE trial conducted across 27 hospitals in China screened a total of 1094 patients with locally advanced G/GEJ adenocarcinoma, of whom 1022 (93%) were enrolled. Among the enrolled patients, 345 (34%) were assigned to the adjuvant CapOx group (8 postoperative cycles), 340 (33%) to the adjuvant SOX group (8 postoperative cycles), and 337 (33%) to the perioperative SOX group (3 preoperative cycles plus 5 postoperative cycles). The study results demonstrated that for patients with LAGC who underwent D2 gastrectomy, the perioperative SOX regimen yielded a clinically meaningful improvement compared with the adjuvant CapOx regimen; additionally, the adjuvant SOX regimen was non‐inferior to the adjuvant CapOx regimen [24]. Therefore, according to a large number of clinical results at home and abroad, platinum combined with fluorouracil chemotherapy regimen (such as SOX, XELOX, FOLFOX, FOLT, etc.) has become the recommended NAC regimens in many Asian countries [14, 24, 25, 26, 27, 28, 29]. Furthermore, the ongoing JCOG 2203 (NEO‐JPEG) study in Japan will further confirm the effectiveness and safety of different NAC drugs in patients with GEJ adenocarcinoma [30].

In conclusion, the effectiveness of NAC varies across different regions and populations. However, most studies suggest that for patients with locally advanced G/GEJ adenocarcinoma, preoperative chemotherapy can improve survival rates, reduce tumor staging, increase the rate of surgical resection, and provide survival benefits. The efficacy of NAC is usually evaluated through imaging (CT, MRI) and pathological tumor regression grade (TRG). Studies have shown that TRG is closely related to prognosis, with patients achieving pathological complete response (pCR) generally having a better prognosis [31, 32].

#### 2.1.2 Problems and Challenges

First, accurately predicting efficacy and personalizing treatment remains challenging. Traditional indicators such as tumor size and staging often fail to accurately reflect the degree of pathological remission. Imaging assessments (such as tumor volume reduction) and postoperative pathological results frequently show inconsistencies (such as pseudo‐remission or underestimation of efficacy), complicating the formulation of personalized preoperative treatment plans. Some studies indicate that not all GC patients benefit from NAC [33, 34]. For instance, a survival analysis of 613 patients in Sweden who underwent curative surgery for GC and survived more than 5 years showed that NAC did not improve long‐term survival in gastric gland cancer survivors. This analysis suggests that the survival advantage may be attributed to the selection of patients with better overall conditions rather than the chemotherapy itself, leading to confounding bias in the study results [35].

Second, regional differences and limitations in treatment regimens present significant challenges. NAC strategies vary globally due to differences in population response, drug availability, and tolerability of side effects. For example, the FLOT regimen is more commonly used in Europe and the United States, while the SOX/XELOX regimen is preferred in Asia. Some studies have shown that NAC does not significantly improve long‐term survival, which may be related to patient selection bias or limitations of the chemotherapy regimen itself. Further research is needed to verify the applicability of different regimens to specific populations.

Additionally, the side effects of chemotherapy and treatment adherence are critical concerns. Common side effects include bone marrow suppression, gastrointestinal reactions (nausea, vomiting), and hair loss, which severely impact the quality of life and may even lead to treatment interruption. The tolerability of chemotherapy is influenced by factors such as age, physique, and comorbidities, necessitating a balance between efficacy and safety.

The timing and duration of treatment are also subjects of debate. A too‐short course may fail to achieve the desired effect, while a too‐long course may increase side effects and the risk of resistance. The optimal treatment duration remains unsettled. There is no consensus on whether to initiate chemotherapy immediately after diagnosis or after stabilizing the patient's condition. Decisions should be made dynamically based on individual patient circumstances.

Therefore, it is particularly important to identify the subpopulations that will benefit most from NAC and to determine appropriate chemotherapy regimens. Some studies have demonstrated that predictive models using machine learning and artificial intelligence can identify advantageous populations, enabling more precise diagnosis and treatment plans [36, 37, 38].

### 2.2 Neoadjuvant Radiotherapy

Radiotherapy can directly kill tumor cells and enhance the effectiveness of chemotherapy, thereby improving the local control rate of tumors. Neoadjuvant radiotherapy (NAR) is particularly suitable for patients unable to undergo radical surgery or those with poor resectability. NAR can improve tumor resectability and is also an important treatment option for patients with tumors in special locations or those resistant to chemotherapy. NAR is commonly combined with NAC in the treatment of G/GEJ adenocarcinoma.

#### 2.2.1 Research Status

In the United States, the proportion of GC surgeries following neoadjuvant chemoradiotherapy (NCRT) is increasing annually, making it a common treatment option [39]. The INT‐0116 study conducted in the United States enrolled a total of 559 patients with GC whose primary tumors were ≥ T3 and/or node‐positive. After R0 resection, these patients were randomly assigned to either the observation group (surgery alone) or the chemoradiotherapy group (postoperative radiotherapy combined with chemotherapy). The chemotherapy regimen consisted of fluorouracil and leucovorin, and all sites at high risk of locoregional recurrence were irradiated to a dose of 45 Gy. The results demonstrated that the chemoradiotherapy group achieved significantly better outcomes compared with the surgery‐alone group [40]. In the study on NCRT for adenocarcinoma of the gastroesophageal junction (AGEJ) conducted by P. van Hagen et al. a total of 368 patients were enrolled, with 366 included in the final analysis: 275 cases (75%) were adenocarcinoma, 84 cases (23%) were squamous cell carcinoma, and 7 cases (2%) were large cell undifferentiated carcinoma. Among all patients, 178 were randomly assigned to the NCRT group and 188 to the surgery‐alone group. Patients in the NCRT group received 5 weeks of chemotherapy with carboplatin plus paclitaxel, concurrent with radiotherapy (total dose of 41.4 Gy in 23 fractions, 5 days per week), followed by surgical resection. The results showed that 92% of patients in the NCRT group achieved R0 resection, compared with 69% in the surgery‐alone group (*p* < 0.001). Among 161 patients who completed NCRT, 47 cases (29%) obtained pathological complete response (pCR). Additionally, postoperative complications were similar between the two groups, with an in‐hospital mortality rate of 4% in both. The median OS was 49.4 months in the NCRT group versus 24.0 months in the surgery‐alone group, indicating a significantly longer OS in the NCRT group (*p* = 0.003) [41]. In a study involving 24 patients who received preoperative short‐course chemoradiotherapy (CRT), all patients underwent CRT (30 Gy in 10 fractions, concurrent with capecitabine or 5‐FU), followed by 2 months of systemic therapy and subsequent surgical resection. All patients completed CRT. After CRT, 22 patients (92%) received chemotherapy, 1 patient (4%) with a microsatellite instability‐high (MSI‐H) tumor underwent immunotherapy, and 1 patient (4%) proceeded directly to surgical resection without systemic therapy. Gastrectomy was performed in 20 patients (83%), with an R0 resection rate of 95%. Two patients achieved pCR, and an additional 5 patients had residual viable tumor ≤ 1%. Surgical complications occurred in 3 patients (1 case (4%) of grade 1, 1 case (4%) of grade 3b, and 1 case (4%) of grade 4a). The 1‐year and 3‐year OS rates were 96% and 85%, respectively. This study demonstrated that this approach is safe and promising for patients with resectable gastric/gastroesophageal junction adenocarcinoma [42]. Furthermore, in a study involving 34 resectable localized gastric adenocarcinoma patients from three medical institutions, 1 patient was excluded. During treatment, patients received up to two cycles of induction chemotherapy (5‐FU, leucovorin, and cisplatin), followed by radiotherapy (45 Gy) with concurrent fluorouracil. The results showed that 28 out of 33 patients (85%) underwent surgery, with an R0 resection rate of 70% and pCR rate of 30%. Eight patients (24%) achieved pathological partial response (MPR, residual primary tumor < 10%). Endoscopic ultrasound (EUS)‐determined T and N stages showed significant downstaging compared with postoperative pathological T and N stages (*p* < 0.01). The median OS of the 33 patients was 33.7 months. Patients who achieved pCR or MPR had a significantly longer median OS than those who did not (63.9 vs. 12.6 months; *p* = 0.03). Two treatment‐related deaths occurred. The study data demonstrated that this three‐step strategy of preoperative induction chemotherapy combined with chemoradiotherapy yields significant pathological responses and confers durable survival benefits [43]. An analysis of data from the Munich Cancer Registry (MCR) on NAC and NCRT for GEJ adenocarcinoma showed that NCRT offers advantages in local disease control, though no significant difference in OS was observed [44]. Results from a phase II trial (RTOG 9904) on NCRT in locally advanced gastric adenocarcinoma patients reported pCR and R0 resection rates of 26% and 77%, respectively, with higher survival rates among 1‐year pCR patients during follow‐up [45]. However, the CALGB 80101 (Alliance) study enrolled 546 patients, who were treated with CRT + surgery + postoperative chemotherapy. Patients were randomized to receive either the FU plus LV regimen or ECF multi‐drug regimen. The results demonstrated that the combination of neoadjuvant radiotherapy (NAR) with the multi‐drug ECF regimen did not confer long‐term survival benefits [46].

In 2024, results from the Korean ARTIST trial presented at the American Society of Clinical Oncology (ASCO) meeting showed a negative trial outcome. There was no significant difference in DFS between the single‐agent XP (capecitabine + cisplatin) and XRT (capecitabine + cisplatin combined with radiotherapy) groups; however, XRT significantly prolonged DFS in Lauren subtypes (intestinal type). Additionally, after a longer follow‐up period, XRT seemed beneficial for patients with lymph node‐positive (LN(+)) GC [47].

A prospective phase II clinical trial in China evaluated the safety and effectiveness of NCRT combined with neoadjuvant consolidative chemotherapy (NCCT) and surgical treatment for locally advanced G/GEJ adenocarcinoma. Patients received NCRT (45 Gy/25 fractions) along with S‐1, followed by NCCT with the SOX regimen for 4–6 cycles, 2–4 weeks after NCRT. Radical surgery was subsequently performed. A total of 46 patients were recruited, with 28 (60.9%) undergoing surgery. All cases (100%) achieved R0 resection, with 20 cases (71.4%) staged as ypT0‐2 and 10 cases (35.7%) showing pCR. Therefore, this study suggests that NAT with NCRT + NCCT + surgery is a feasible option for locally advanced G/GEJ adenocarcinoma, characterized by a low incidence of severe adverse reactions and good efficacy [48].

A phase III international trial [49] included 574 patients from 70 centers in Australasia, Canada, and Europe. Patients with resectable G/GEJ adenocarcinoma were randomly assigned to receive either preoperative chemotherapy plus perioperative chemotherapy or perioperative chemotherapy alone. In both groups, patients received ECF or FLOT before and after surgery. The preoperative chemotherapy group also received radiotherapy (45 Gy over 25 fractions plus fluorouracil infusion). The results showed that preoperative chemotherapy plus perioperative chemotherapy did not improve OS compared to perioperative chemotherapy alone. However, the proportion of patients achieving pCR (17% vs. 8%) and downstaging after surgery was higher in the preoperative chemotherapy group. No significant differences in OS or PFS were observed at a median follow‐up of 67 months. Treatment‐related toxicities were similar in both groups [49].

A retrospective analysis of 14,266 patients from the National Cancer Database (NCDB) in the United States showed that NAC had better outcomes than NCRT. However, a better AJCC stage after postoperative neoadjuvant chemoradiotherapy may lead to an overestimation of the survival rate [50]. American scholars led by Grace Lee summarized that preoperative radiotherapy may promote tumor downstaging and improve R0 resection, thereby better controlling the local region and improving survival. However, the role of preoperative chemoradiotherapy in resectable GC remains unclear, with no randomized data available to date, although phase II single‐arm studies have shown promising results [51].

Based on previous studies, NCRT has shown consistent improvement in R0 resection rates, pCR occurrence rates, and reliable safety, with varying conclusions on DFS and OS.

#### 2.2.2 Problems and Challenges Faced

Although the NCRT shows significant efficacy in locally advanced G/GEJ adenocarcinoma (such as improving tumor downstaging and R0 resection rates), the accompanying toxic side effects cannot be ignored. Acute complications such as radiation esophagitis, gastritis, and bone marrow suppression may force patients to discontinue treatment. For example, in the RTOG9904 study, some cases had to terminate treatment due to toxicity [45]. Long‐term surgical complications (such as anastomotic leakage and intestinal obstruction) also need to be considered. Patient tolerance differences (such as age, nutritional status, chronic diseases, etc.) further complicate individualized risk–benefit assessment.

The precision of radiotherapy technology faces challenges. Factors such as respiration and peristalsis can cause dynamic displacement of the stomach, requiring image guidance (4D‐CT) or adaptive radiotherapy technology to optimize target dose distribution. Although IMRT and VMAT have improved conformality, completely overcoming target area omission or normal tissue damage due to organ motion remains difficult. Proton/heavy ion therapy, while theoretically reducing dose to surrounding organs, is limited in application due to high costs and lack of clinical evidence.

Additionally, the synergistic mechanism and dose optimization of combined treatment regimens remain challenging. The synergistic mechanisms of radiotherapy and chemotherapy or immunotherapy are not yet clear, and there is no consensus on the timing of drug use (synchronous/sequential) and dose fractionation patterns (conventional/large fraction). For example, although the OGSG1303 phase I trial explored the recommended dose of S‐1 combined with radiotherapy, the risk of overlapping toxicity still needs further validation [52].

The timing of treatment and its connection with surgery are controversial. There is no unified standard for the course of preoperative radiotherapy (2–5 weeks) and the interval between radiotherapy and surgery (4–6 weeks). A prolonged interval may increase the difficulty of surgery due to tissue fibrosis, while a shorter interval may affect tumor regression. Additionally, radiotherapy may increase the risk of intraoperative bleeding and delay anastomosis healing postoperatively, requiring a balance between local control and surgical safety.

### 2.3 Neoadjuvant Immunotherapy

In recent years, immune checkpoint inhibitors (ICIs) have made significant strides in tumor immunotherapy, exhibiting potent anti‐tumor activity in various solid tumors such as melanoma, non‐small cell lung cancer, renal cancer, and prostate cancer. Recognized as one of the most important scientific breakthroughs of 2013 by the journal *Science*, tumor immunotherapy has shown outstanding efficacy and innovation. Studies such as ATTRACTION‐2, CheckMate‐032, CheckMate‐649, and KEYNOTE‐062 have demonstrated that immunotherapy is effective in treating advanced GC. Notable advancements have been achieved with new immune therapies targeting Programmed Cell Death Protein‐1 (PD‐1) and its ligand (PD‐L1) in the treatment of advanced GC. However, the efficacy of NAT still requires further large‐scale and long‐term evaluation. Treatment strategies for locally advanced G/GEJ adenocarcinoma in the neoadjuvant stage are evolving, moving from simple chemotherapy to combinations of chemotherapy and immunotherapy, as well as chemotherapy combined with immunotherapy and targeted therapy. Postoperative treatment strategies are also continually being updated. Chemotherapy combined with immunotherapy has become a focal point in current research on G/GEJ adenocarcinoma, with some mid‐term data analyses showing promising clinical outcomes [53, 54]. Consequently, some countries have included it as a neoadjuvant treatment option.

#### 2.3.1 Research Status

##### 2.3.1.1 Research Status in China

In 2022, a Phase 2 study enrolled 36 patients with resectable G/GEJ adenocarcinoma at clinical stage cT3‐4NanyM0, among whom 7 had GEJ cancer and 34 were at clinical Stage III. The sintilimab plus CapeOx regimen was given as NAT (3 cycles preoperatively), followed by 3 cycles of adjuvant therapy with the same‐dose CapeOx regimen after surgery. The results showed that 35 patients completed the NAT, and all patients underwent surgery with an R0 resection rate of 97.2%. Additionally, 19.4% of the patients achieved pCR and 47.2% attained MPR. The 1‐year DFS rate and OS rate were 90.3% and 94.1%, respectively. This regimen presented a favorable safety profile: the most common grade 3 treatment‐related adverse events were anemia and neutropenia (5 cases each, 13.9%), and no serious adverse events or grade 4–5 adverse events were observed [55]. In the same year, a prospective, single‐arm, open‐label Phase 2 trial enrolled 32 patients, who received NAT with the PD‐1 inhibitor tislelizumab combined with the SOX regimen (planned for 3 cycles, with 1 patient completing 2 cycles). The results showed that 53.1% of the patients achieved MPR and 25.0% attained pCR; the 1‐year OS rate was 91.4% and the 1‐year RFS rate was 90.0%. During the neoadjuvant treatment period, 65.6% of the patients experienced adverse events, with 12.5% being grade III‐IV [56].

At the 2023 Annual Meeting of the American Society of Clinical Oncology (ASCO), the team led by SQ Yuan from Sun Yat‐sen University Cancer Center presented the findings of the randomized, open‐label Phase 2 NEOSUMMIT‐01 trial. A total of 108 patients with resectable G/GEJ cancer at clinical stage cT3‐4aN + M0 were enrolled in the trial and randomized 1:1 into two groups with 54 patients in each. The chemotherapy group received the SOX/XELOX regimen (once every 3 weeks), with 3 preoperative cycles and 5 postoperative cycles completed. The toripalimab plus chemotherapy group was administered the same SOX/XELOX regimen in combination with the PD‐1 inhibitor toripalimab, followed by toripalimab monotherapy maintenance for up to 6 months postoperatively. The results confirmed that the toripalimab plus SOX/XELOX regimen was significantly superior to chemotherapy alone in efficacy: the rates of TRG 0/1 (44.4% vs. 20.4%, *p* = 0.009) and pCR (22.2% vs. 7.4%, *p* = 0.030) in the combination therapy group were both significantly higher, and the prespecified study endpoint was achieved. In terms of safety, the two groups had similar incidences of surgical complications, mortality and grade 3–4 treatment‐related adverse events, indicating a manageable safety profile of this combination regimen [57]. A study analyzing adverse events in 6692 patients from 20 studies found that those receiving immunotherapy had a higher incidence of treatment discontinuation due to adverse events, severe adverse events, and grade 3 or higher adverse events compared to chemotherapy patients [58]. A retrospective study collected 24 patients with resectable GEJ adenocarcinoma treated at Jining First People's Hospital from October 2020 to October 2023, all of whom received NAT with sintilimab combined with chemotherapy followed by surgical treatment. The results showed that the pCR rate of the patients was 16.7% with a 100% R0 resection rate; the treatment‐related adverse events were manageable, and the most common postoperative complication was pneumonia (45.8%). The median OS was not reached at a median follow‐up of 13.5 months, and the long‐term efficacy of this regimen remains to be confirmed by further follow‐up [59].

In 2024, the results of the phase II Wuhan UHGI001 trial were released. This study enrolled 49 patients with cT3/4aN + M0 locally advanced G/GEJ adenocarcinoma, who received 3 cycles of NAT with tislelizumab combined with the SOX regimen preoperatively and 5 cycles of postoperative adjuvant SOX chemotherapy. The results showed that 49.0% of the patients (24/49) achieved MPR and 26.5% (13/49) attained pCR. With a median follow‐up of 26.8 months, the 2‐year PFS rate and OS rate of the patients were 69.4% and 81.2%, respectively. In this study, the incidence of grade 3–4 adverse events was 12.2% during the neoadjuvant treatment phase, 17.0% postoperatively, and 15.2% during the adjuvant treatment phase [60]. A single‐arm phase II clinical trial enrolled 29 patients with locally advanced resectable G/GEJ adenocarcinoma from September 2020 to January 2022. All patients received 3 cycles of NAT with camrelizumab combined with the SOX regimen (once every 3 weeks), followed by surgical resection and adjuvant therapy with the same regimen. All patients in this study completed the NAT and underwent surgery. The results showed that the rates of pCR and total pathological complete response (tpCR, ypT0N0) both reached 10.3% (3/29), MPR rate was 69.0% (20/29), and the R0 resection rate was 96.6% (28/29). In terms of safety, 82.8% of the patients experienced treatment‐emergent adverse events (TEAEs) of any grade, 44.8% had immune‐related adverse events (irAEs) of any grade, and no grade 3 or higher adverse events were reported in this trial [61]. A multicenter Phase II study on neoadjuvant Cadonilimab combined with FLOT chemotherapy reported that among 38 enrolled patients, 32 completed the planned treatment. The study achieved a 100% R0 resection rate (32/32), a 21.1% pCR rate (8/38), and a 44.7% MPR rate (17/38). Independent central imaging review included 28 of the 38 patients, with an ORR of 60.7% (17/28) and DCR of 100.0% (28/28). Tumor stage reduction was observed in 71.9% of patients (23/32), with consistent efficacy across subgroups. In terms of safety, 31.6% of patients experienced grade 3 adverse events, but no serious safety issues were reported [62].

A multicenter retrospective study found that neoadjuvant SOX plus ICI was associated with better tumor regression compared to SOX alone in treating LAGC, with similar TRAEs. SOX + ICIs proved safe and feasible for laparoscopic GC surgery, with reduced estimated blood loss [63]. A study by Bin Wang's team from the Army Medical University of China demonstrated that, after balancing baseline characteristics using propensity score matching, the NAC immunotherapy (NAIC) group had a significantly higher pCR rate (25%) than the NAC (3.8%), with 43% of patients in the NAIC group achieving a MPR compared to 17% in the NAC group [64]. These studies suggest that NAIC may enhance pathological response in patients with LAGC, potentially offering greater survival benefits. A propensity score‐matched analysis of LAGC patients from 47 hospitals in the National Cancer Information Database of China (January 2019 to December 2022) demonstrated that neoadjuvant ICIs combined with chemotherapy improved the pCR rate and 2–3 year DFS rate compared to chemotherapy alone. However, it remains uncertain whether these short‐term benefits translate into long‐term efficacy [65]. The triple regimen did not show superiority over the dual regimen in terms of efficacy. The combination with immunotherapy exhibited comparable postoperative safety. The ongoing NCT06482788 study aims to validate the efficacy and safety of PD‐L1 inhibitor (Adebrelimab) combined with XELOX chemotherapy in the perioperative treatment of resectable GEJ adenocarcinoma patients [66].

##### 2.3.1.2 Research Status Abroad

The interim results of the global, randomized, double‐blind, placebo‐controlled Phase III MATTERHORN study led by a U.S. research team in 2022 demonstrated that Durvalumab, in combination with FLOT chemotherapy, may enhance anti‐tumor activity in patients with resectable G/GEJ adenocarcinoma [67, 68]. In 2023, WJ Sun from the Department of Oncology at the University of Kansas Medical Center evaluated the efficacy and tolerability of mFOLFOX6 combined with Pembrolizumab in a Phase II single‐arm study involving patients with resectable G/GEJ adenocarcinoma. Among the 26 patients who underwent planned curative surgery, all achieved R0 resection, and 16% exhibited a pCR; 54% experienced grade 3 or higher TRAE, with no unexpected toxicities observed during the perioperative period. The combination therapy of Pembrolizumab and mFOLFOX for resectable G/GEJ adenocarcinoma was highly effective, achieving a pCR of 21% and providing significant long‐term survival benefits [69]. However, the ATTRACTION‐5 study, which evaluated the use of Nivolumab in combination with chemotherapy as adjuvant therapy for pathologic stage III G/GEJ adenocarcinoma in Japan, Korea, Taiwan, and mainland China, did not meet the primary endpoint of recurrence‐free survival (RFS) and is not currently recommended [70]. The latest data from the 2024 ASCO Gastrointestinal Cancers Symposium showed that adding Durvalumab to FLOT in the perioperative period significantly improved pCR. However, it remains unclear whether this improvement will translate into better EFS and OS outcomes. In the phase II segment of the multicenter randomized DANTE trial, 295 patients with resectable GEJ adenocarcinoma at stage ≥ cT2 or cN+ were enrolled and randomized 1:1 into two groups. Group A received 4 cycles of the FLOT regimen combined with atezolizumab during the perioperative period plus an additional 8 cycles of atezolizumab maintenance therapy, while Group B was administered the perioperative FLOT regimen alone. The results showed no significant differences between the two groups in surgical morbidity, 60‐day mortality, R0 resection rates (96% vs. 95%) or adverse event profiles. Nevertheless, Group A achieved significantly superior tumor downstaging efficacy (ypT0: 23% vs. 15%; ypN0: 68% vs. 54%) and a higher pCR rate (24% vs. 15%), with this therapeutic benefit being more pronounced in the PD‐L1 CPS ≥ 10 (33% vs. 12%) and MSI subgroups (63% vs. 27%). This study confirmed that perioperative FLOT combined with atezolizumab is safe and feasible for patients with resectable EGJ adenocarcinoma, and can significantly improve tumor pathological downstaging and histopathologic regression outcomes [71]. Dr. Kohei Shitara from the National Cancer Center Hospital in Japan led the mid‐term results of the KEYNOTE‐585 study, and the final result demonstrated that neoadjuvant/adjuvant Pembrolizumab combined with FLOT is feasible in the perioperative treatment of locally advanced G/GEJ adenocarcinoma and showed a trend towards improved OS for patients [72, 73, 74]. The phase II PANDA trial enrolled 21 patients with previously untreated, resectable, non‐metastatic G/GEJ cancer, who received a neoadjuvant regimen of 1 cycle of atezolizumab monotherapy followed by 4 cycles of atezolizumab combined with docetaxel, oxaliplatin and capecitabine. This regimen was well tolerated, with only 2 patients (10%) developing grade 3 irAEs and no grade 4 or 5 events reported; all patients underwent uneventful surgical resection without treatment‐related delays, meeting the primary endpoint of safety and feasibility. Among the 20 patients who completed surgery, 70% (14/20) achieved MPR and 45% (9/20) attained pCR. With a median follow‐up of 47 months, the responders had a markedly superior survival outcome: 13 of 14 responders remained alive and disease‐free, while 5 of 6 non‐responders died of tumor recurrence [75].

##### 2.3.1.3 Current Application Status of ICIs in Special Populations

Neoadjuvant immunotherapy has shown remarkable efficacy in patients with high PD‐L1 expression or high microsatellite instability (MSI‐H), leading to an early recommendation of immunotherapy in the neoadjuvant treatment stage. A high‐quality article published by the research team led by LH Meng in China pointed out that MSI is caused by defects in DNA mismatch repair (MMR) protein function, closely related to the occurrence, development, prognosis, and efficacy prediction of various tumors. MSI‐H and deficiency in mismatch repair proteins (dMMR) may be good prognostic factors for GC patients and may also be negative predictive factors for the effectiveness of adjuvant chemotherapy in resectable GC. MSI‐H/dMMR is also a predictive biomarker for the effective treatment of ICIs in advanced GC [76].

For dMMR patients, perioperative immunotherapy is a promising future development. A study conducted in 2022, which included an Asian patient population, further confirmed the efficacy of this approach in such patients [77]. In 2023, the GERCOR NEONIPIGA study and the INFNITY study explored the use of Tremelimumab combined with Durvalumab as NAT for resectable G/GEJ adenocarcinoma with MSI, dMMR, and Epstein–Barr virus (EBV) negativity, demonstrating a pCR rate of 60%. This provided new evidence of efficacy for locally advanced G/GEJ adenocarcinoma patients with MSI and dMMR. The studies revealed that NAT with PD‐1/PD‐L1 antibodies combined with cytotoxic T‐lymphocyte‐associated protein 4 (CTLA‐4) antibodies achieved pCR rates of 59% and 60%, respectively [78, 79]. The IMHOTEP study on perioperative immunotherapy for MSI/dMMR tumors showed a pCR of 25% with Pembrolizumab [80]. Additionally, the DANTE study confirmed the efficacy of Atezolizumab combined with chemotherapy in tumor downstaging and improving pCR rates, with a pCR of 63% in MSI‐H patients. This subgroup analysis demonstrated the high efficacy of immunotherapy in MSI‐H patients, but long‐term survival data are lacking, and it cannot yet be recommended as a routine clinical treatment [81]. A retrospective 4.5‐year study conducted by the Napalkov Cancer Center in 2024 on locally advanced MSI‐H GC patients showed that ICIs as NAT for MSI‐H LAGC are safe and effective [82].

In summary, related clinical studies of NAC combined with ICIs for locally advanced G/GEJ adenocarcinoma have been successively published domestically and internationally. According to existing research, ICIs combined with chemotherapy have shown good effects in disease control, pCR, and DFS, but there was a significant difference in the presence of severe complications during treatment [83, 84]. Similarly, some research results still indicate that serious complications should not be underestimated. However, due to the lack of large sample data supporting the 5‐year survival related to these diseases, only short‐term data is available. The mid‐term study results of ICIs in locally advanced G/GEJ adenocarcinoma show good clinical efficacy, especially in special populations such as dMMR, MSI‐H, and EB virus, which have significant benefits. Some studies suggest that ypN0 may be an important substitute for favorable clinical outcomes in LAGC patients receiving NICT plus radical surgery [85]. However, current research has a small sample size and lacks long‐term survival data.

#### 2.3.2 Problems and Challenges Faced

Firstly, substantial individual differences exist in the efficacy of immunotherapy. The effectiveness of treatments like PD‐1/PD‐L1 inhibitors is influenced by various factors, including the tumor microenvironment (such as immune cell infiltration, antigen presentation capability), genetic background, and gut microbiota (such as a higher abundance of lactobacilli correlates with better efficacy [86]). These variations can result in a lack of response or primary resistance in some patients. Currently, there is an absence of precise biomarkers to identify responsive populations accurately [60]. Although studies indicate that intestinal‐type gastric adenocarcinoma may be more sensitive to immunotherapy, further research into universal biomarkers (such as molecular subtypes and immune scores) is necessary [87, 88].

Secondly, managing immune‐related adverse events (irAEs) is complex. Immunotherapy can induce multi‐organ toxicities (such as pneumonitis, thyroid dysfunction, myocarditis) with unpredictable onset and severity [89, 90]. For instance, immune pneumonitis can rapidly progress to respiratory failure, and myocarditis can be fatal, necessitating long‐term monitoring. Unlike traditional chemotherapy, irAEs require targeted interventions (such as steroid therapy) and may affect treatment continuity. Early identification, accurate grading, and balancing efficacy with safety are critical challenges in clinical practice [91, 92].

### 2.4 Neoadjuvant Targeted Therapy

#### 2.4.1 Research Status

Targeted therapy for advanced GC has shown success in specific populations, but its application in the neoadjuvant setting is still being explored. Current small‐scale studies have demonstrated its efficacy.

In 2017, a multicenter study conducted in Italy demonstrated that neoadjuvant XELOX chemotherapy combined with Trastuzumab can improve the pCR rate in patients with human epidermal growth factor receptor 2 (HER2)‐positive status [93]. In 2020, the PETRARCA trial compared neoadjuvant Trastuzumab and Pembrolizumab with FLOT chemotherapy versus FLOT alone in HER2‐positive resectable GEJ cancers. Results showed that adding Trastuzumab and Pertuzumab to FLOT significantly increased pCR and lymph node‐negative rates, despite more frequent side effects such as diarrhea and leukopenia [94, 95]. The 2021 HER‐FLOT study confirmed that Trastuzumab with FLOT also improved pCR and R0 resection rates in advanced GEJ cancers [96].

Beyond HER2, targeted therapies against VEGFR and EGFR have been explored in neoadjuvant GC treatments. Apatinib, a VEGFR inhibitor, has shown promise in phase II studies, improving tumor shrinkage and surgical outcomes in LAGC. A prospective cohort study enrolled 96 patients with LAGC, who were divided into the apatinib plus chemotherapy group (*n* = 45) and the chemotherapy‐alone group (*n* = 51) based on their treatment of choice. Both groups received 3 preoperative cycles of chemotherapy with either the SOX or CapOx regimen, with the apatinib plus chemotherapy group additionally receiving oral apatinib at a daily dose of 375 mg. The results showed that ORR of the apatinib plus chemotherapy group (62.2%) was significantly higher than that of the chemotherapy‐alone group (37.3%), with a more favorable pathological response grade as well. Although pCR rate (23.3% vs. 9.3%) and R0 rate (95.6% vs. 84.3%) in the apatinib plus chemotherapy group showed an increasing trend, no statistically significant differences were observed in these two indices between the two groups [97].

Ramucirumab, another VEGFR‐2 inhibitor, is being evaluated for its potential in neoadjuvant settings to reduce tumors and improve survival [98]. Cetuximab, targeting EGFR, showed limited efficacy in some studies but may enhance chemotherapy or radiotherapy responses. A study involving 65 patients with resectable G/GEJ adenocarcinoma administered 6 cycles of perioperative chemotherapy with cetuximab combined with cisplatin and simplified LV5FU2 before and after surgery. The results showed that only 29.7% of the patients achieved radiological OR, 95.3% did not discontinue treatment due to major toxicity, 28.5% of the 56 evaluable patients attained histological response (Mandard tumor regression grade ≤ 3), and 92.3% of the patients completed surgical resection. With a median follow‐up of 44.5 months, the median DFS of the patients was 24.4 months and the median OS was 40.3 months. This study confirmed that the combination of cetuximab and the aforementioned chemotherapy regimen had a good safety profile, but its efficacy failed to meet the predefined primary endpoint [99]. These studies highlight the potential of targeted therapies in shaping personalized, molecularly tailored treatments for GC. Additionally, ongoing research into combining targeted drugs with chemotherapy is expected to expand treatment options. Fruquintinib, a VEGFR inhibitor, is being tested in a phase II trial (FRUTINEOGA) for the NAT of locally advanced G/GEJ adenocarcinoma, with promising early outcomes expected for tumor regression and survival [100].

#### 2.4.2 Problems and Challenges Faced

First, drug resistance is prominent. In HER2‐positive GC patients, progression often occurs within several months after Trastuzumab use due to tumor heterogeneity and resistance mechanisms (such as HER2 signal loss and bypass activation). There is a lack of effective treatment after drug resistance, and there is an urgent need to develop new drugs (such as antibody‐drug conjugates (ADCs)) or combined immunotherapy strategies.

Secondly, the target population is narrow. Existing targeted drugs cover only about 20% of patients (HER2‐positive or VEGFR targets), with the majority of HER2‐negative or non‐driver gene mutation patients lacking options. The identification of new targets (such as Claudin18.2) through multi‐omics analysis is needed to expand the beneficiary population.

Furthermore, combination therapy poses challenges. When targeted therapy is combined with chemotherapy or immunotherapy, the risk of cumulative toxicity is significant (such as Trastuzumab combined with anthracyclines increasing cardiac toxicity and anti‐VEGFR drugs exacerbating bleeding). The optimal drug sequence, dosage, and safety management need to be confirmed by large‐scale clinical trials.

Finally, there is a lack of biomarkers. Apart from HER2, the lack of precise predictive biomarkers results in ineffective treatment for some patients. It is necessary to integrate ctDNA, tumor microenvironment characteristics (such as PD‐L1 expression), and radiomics to build a dynamic predictive model.

### 2.5 Neoadjuvant Multimodal Therapy

Due to the heterogeneity of GC, single therapy is prone to drug resistance, whereas multimodal combination therapy can synergistically inhibit various signaling pathways (such as HER2, VEGF, and PD‐1/PD‐L1). Currently, multimodal combination therapy has shown promising results in advanced GC, laying the foundation for NAT [101]. Neoadjuvant multimodal combination therapy aims to increase the R0 resection rate, achieve pCR or significant downstaging, and ultimately prolong OS and DFS. Therefore, multimodal combination therapy may become a focal point of future research [102].

#### 2.5.1 Research Status

##### 2.5.1.1 Research Status in China

The Neo‐PLANET II phase II trial of NAC + radiotherapy + immunotherapy in locally advanced G/GEJ adenocarcinoma demonstrated a pCR rate of 33.3%, with total pCR, MPR, and R0 resection rates of 33.3%, 44.4%, and 91.7%, respectively. Among the 28 patients, 77.8% achieved ypN0. The 2‐year PFS and OS rates were 66.9% and 76.1%, respectively. The most common grade 3–4 adverse event was a decrease in lymphocyte count (27 cases, 75.0%). The study showed a favorable pCR rate and safety profile [103]. The multicenter, randomized phase III DRAGON IV/CAP 05 trial focused on patients with T3‐4aN + M0 locally advanced G/GEJ adenocarcinoma. Patients were initially randomized 1:1:1 into three arms: the SOXRC arm (camrelizumab plus low‐dose rivoceranib and SOX), the SOXR arm (high‐dose rivoceranib plus SOX), and the SOX‐alone arm. Enrollment in the SOXR arm was subsequently terminated due to safety concerns, and subsequent patients were randomized 1:1 into the SOXRC arm and the SOX‐alone arm. This study reported data on the first 360 surgically eligible patients from the two arms. The results showed that pCR rate in the SOXRC arm (18.3%) was significantly higher than that in the SOX‐alone arm (5.0%). Additionally, the SOXRC arm had a lower surgical complication rate (27% vs. 33%). Although the incidence of grade ≥ 3 neoadjuvant treatment‐related adverse events was slightly higher in the SOXRC arm (34% vs. 17%), the overall safety profile was tolerable. These findings confirm that the SOXRC regimen demonstrates a significant advantage in improving patients' pathologic response [104]. In a retrospective study of 38 patients, neoadjuvant SOX regimen chemotherapy combined with anti‐angiogenesis therapy (particularly Apatinib) and ICI treatment for LAGC achieved an R0 resection rate of 97.4%. The MPR rate was 47.4%, with a pCR rate of 23.7%. The 1‐year OS rate was 100%, and the DFS rate was 94.7%. Neoadjuvant treatment was generally well tolerated, with no grade 5 TRAEs reported; only one patient experienced a grade 4 TRAE (immunotherapy‐related hepatitis) [105]. Furthermore, a prospective, single‐arm, phase II study on neoadjuvant Sintilimab and Apatinib combined with perioperative FLOT chemotherapy for LLAGC showed that 33.3% of patients experienced at least one grade III or IV TRAE during the NAT period. The combination therapy group had a pCR rate of 28.6%. The 1‐year DFS rate was 92.6%, and the 18‐month cumulative DFS rate was 78.9%. The 1‐year OS rate was 96.4%, with a cumulative 2‐year OS rate of 84.4% [106].

##### 2.5.1.2 Research Status Abroad

In a phase Ib trial conducted by a US research team, neoadjuvant Nivolumab or Nivolumab + LAG‐3 inhibitor Relatlimab combined with chemoradiotherapy was evaluated in 32 resectable stage II/III G/GEJ adenocarcinoma patients. Group A showed pCR and MPR rates of 40% and 53.5%, respectively, while Group B had rates of 21.4% and 57.1%. Common adverse events included fatigue, nausea, thrombocytopenia, and dermatitis. Overall, the 2‐year RFS and OS rates were 72.5% and 82.6%, respectively [107].

In summary, the multimodal approach of immunotherapy + targeted therapy + chemotherapy/radiotherapy significantly improves pCR and survival rates. Early clinical trials of second‐generation immune checkpoint inhibitors such as LAG‐3 and TIGIT are in progress. The CROSS regimen remains a global standard for LAGC. Although multimodal combination therapy has achieved some success, the current limitation is the lack of large sample size support [108]. Several relevant studies are ongoing, including the ChiCTR 2200062285 study investigating the effectiveness and safety of SOX chemotherapy combined with Apatinib and Camrelizumab in NAT for locally advanced G/GEJ adenocarcinoma patients [109], and a prospective, multicenter, single‐arm, Ib/II phase clinical trial (NCT 06266871) studying the effectiveness and safety of NAC combined with Tislelizumab and low‐dose radiotherapy in treating G/GEJ adenocarcinoma patients, which may provide new treatment strategies for this patient population [110].

#### 2.5.2 Problems and Challenges Faced

The timing and optimization of combining immunotherapy with other treatment modalities are still under exploration. The synergistic mechanisms between immunotherapy and chemotherapy, radiotherapy, targeted therapy, etc., when used concurrently, are complex. The optimal combination regimen, drug sequence, and treatment cycle remain undetermined. The toxic side effects may be cumulative during combination therapy, increasing the difficulty of patient tolerance.

The combination strategies lack standardized protocols, and the optimal combinations of chemotherapy, immunotherapy, targeted therapy, and radiotherapy (such as sequence, dosage, and cycles) have yet to be defined. Current clinical staging systems fail to accurately reflect biological behavior, which may result in over‐treatment or under‐treatment for some patients. Certain cT3N0 patients may not benefit significantly from NAT, yet still need to bear the risk of toxicity. Most evidence for combination therapies comes from phase I/II or retrospective studies, with fewer phase III randomized controlled trials (RCTs) and insufficient long‐term survival data. There are discrepancies between international guidelines (e.g., NCCN/ESMO) and domestic guidelines (e.g., CSCO) regarding the selection of neoadjuvant regimens, particularly concerning the indications for chemoradiotherapy.

In summary, the challenges of multi‐modal combination therapy for GC center on toxicity control, efficacy prediction, personalized treatment optimization, and clinical translation. In the future, through technological innovations, precise biomarker stratification, and global collaborative research, these bottlenecks need to be gradually overcome to achieve the goal of “efficient and low‐toxic” personalized treatment.

## 3 Future Development of Neoadjuvant Therapy

Locally advanced G/GEJ adenocarcinoma is expected to undergo significant adjustments in NAT with the development of new drugs and the analysis of extensive clinical trial data. In the field of NAR, research involving heavy ions, among others, holds promise. With the current surgical treatment model, more precise surgical techniques are gradually being adopted, and the advantages of robotic surgery are becoming increasingly evident with advancements in surgical instruments and artificial intelligence [111, 112]. Postoperative adjuvant therapy is expected to improve through the clinical use of new drugs and experiential summaries. Global research provides a platform for discussing the optimal indications for NAC, paving the way for future global cooperation to better treat patients with locally advanced G/GEJ adenocarcinoma [113].

### 3.1 Optimization of Individualized Treatment Strategies

With the advancement of gene sequencing and molecular targeting technologies, individualized and precise treatment strategies will be further refined. The core of precision medicine lies in selecting individualized treatment populations through biomarkers. For example, HER2‐positive patients can benefit from Trastuzumab combined with chemotherapy/immunotherapy, improving the pathological response rate. MSI‐H/dMMR patients, due to their high tumor mutation load, are sensitive to immunotherapy, and using PD‐1 inhibitors in the neoadjuvant stage can significantly improve efficacy. Patients with PD‐L1 CPS ≥ 5 or TPS ≥ 1% and high tumor mutational burden (TMB) are more likely to benefit from immunotherapy combined with chemotherapy, thereby avoiding overtreatment. Integrated multi‐omics technologies (genomics, transcriptomics, proteomics, metabolomics) can identify novel biomarkers (such as drug‐resistance gene combinations), build precise molecular subtypes, and guide the selection of targeted and immunotherapy. The 2023 NCCN Guidelines added neoadjuvant or perioperative immunotherapy regimens for resectable GC patients with MSI‐H/dMMR [114]. Therefore, more biomarkers need to be developed to guide the selection of treatment regimens [115, 116]. For instance, a clinical cohort study of stage III GC patients emphasized that optimizing NAT should focus on the principles of precision medicine, including identifying appropriate patient populations, tailoring personalized treatment plans, and determining the optimal timing for surgery [117].

Moreover, liquid biopsy enables non‐invasive dynamic monitoring by detecting circulating tumor cells (CTC), circulating tumor DNA (ctDNA), and exosomes in the blood. In the future, integrating multidimensional liquid biopsy (CTC + ctDNA + exosomes) can establish a comprehensive management system, combined with point‐of‐care testing (POCT) technology for rapid detection at the grassroots level, promoting the implementation of precision therapy.

### 3.2 Exploration of Integrated Treatment Modes

The efficacy and safety of new adjuvant therapies based on ICIs in locally advanced G/GEJ adenocarcinoma have been further confirmed [118]. The combined application of multiple therapies is expected to improve both cure and survival rates.

#### 3.2.1 Rational Combination of Different Treatment Methods

The combination of chemotherapy and immunotherapy represents a significant breakthrough. Chemotherapy eradicates tumor cells, releases antigens, reduces immune suppressor cells, and enhances the response to immunotherapy. Optimization strategies include selecting synergistic chemotherapy drugs, exploring the sequence of administration with immunotherapy (synchronous or sequential), and accurately selecting the advantageous population based on biomarkers.

The potential synergistic effect of targeted therapy and immunotherapy is noteworthy. For example, combining HER2‐targeted drugs with immunotherapy can enhance immune recognition of tumor cells. Anti‐angiogenic drugs (such as VEGFR inhibitors) improve the immune microenvironment through “vascular normalization” and promote T cell infiltration. However, issues related to dosage ratios, overlapping toxicities (e.g., cardiac toxicity, bleeding risk), and optimal treatment timing need to be addressed. Safety must be confirmed through Phase III clinical trials.

Further, the multidimensional coordination between radiotherapy and immunotherapy merits exploration. Radiotherapy not only directly kills tumor cells but also activates systemic immunity through immunogenic cell death (the “abscopal effect”). Key challenges include balancing radiation dose (high doses damage tissues, low doses may be insufficient), sensitization and immune‐modulating effects of chemotherapy, and the timing of immune therapy intervention (too early leads to tolerance, too late misses the activation window).

Moreover, an interdisciplinary dynamic combination strategy is a future trend. This requires integrating the local–global dual‐strike mode of chemotherapy, targeted therapy, radiotherapy, and immunotherapy while using multi‐omics biomarkers (such as ctDNA, immune microenvironment characteristics, imaging omics) to achieve individualized stratified treatment. Research on optimizing treatment strategies is ongoing to reduce side effects and improve patients' quality of life, with more prospective large‐scale clinical studies expected to provide further confirmation [119].

#### 3.2.2 Precise Adjustment of Treatment Sequence and Cycle

Individualized therapy should be prioritized based on a comprehensive assessment of tumor characteristics and patient status. For example, for HER2‐positive or high tumor burden patients, targeted therapy combined with chemotherapy should be prioritized to shrink the tumor, followed by sequential immune therapy. For MSI‐H or TMB‐H patients, initiating immune therapy to activate the immune response should be prioritized, followed by a combination of radiotherapy and chemotherapy to consolidate efficacy. Elderly or frail patients should choose a milder approach (such as immune or targeted therapy first), gradually adding other treatments. Young or tolerant patients can receive intensified combination therapy (such as targeted + chemotherapy + immune therapy), but adverse reactions must be closely monitored.

The treatment cycle needs to be dynamically adjusted based on real‐time feedback. Evaluate changes in tumor volume and metabolic activity through imaging (PET‐CT/MRI) and dynamically track efficacy by combining levels of blood markers (CEA, CA19‐9, ctDNA). Sensitive patients should complete the cycle as planned to ensure tumor regression; for resistant or progressing patients, treatment regimens should be switched in a timely manner (such as changing targeted drugs or advancing surgery) to avoid delays in ineffective treatment.

Balancing treatment and the timing of surgery is crucial. It is necessary to balance adequate NAT (reducing tumor size, lowering staging) with avoiding overtreatment (such as prolonged chemotherapy cycles leading to delayed surgery) to maximize surgical resection rates and reduce the risk of recurrence.

### 3.3 Development of New Drugs and Optimization of Treatment Strategies

The expansion of immunotherapy drugs has revitalized NAT for GC. Rapid advancements in immune checkpoint inhibitors, targeted drugs, tumor vaccines, and cell therapy are reshaping the treatment landscape of GC, enabling more innovative drugs to enter the treatment regime for locally advanced G/GEJ adenocarcinoma [120].

Emerging immune checkpoint inhibitors targeting novel molecules such as LAG‐3 and TIGIT can synergistically enhance the efficacy of PD‐1/PD‐L1 inhibitors and overcome resistance. For instance, findings from a phase 1b/2 study involving patients with HER2‐negative, unresectable, advanced/metastatic G/GEJ adenocarcinoma demonstrated that no dose‐limiting toxicities were observed during the dose‐escalation phase of phase 1b, with the recommended phase 2 dose set at 6 mg/kg administered once every 2 weeks. The phase 2 study met its primary endpoint of ORR, which stood at 52.1% (4.3% CR, 47.9% PR). mDOR, mPFS, and mOS were 13.73 months, 8.18 months, and 17.48 months, respectively. Notably, significant survival benefits were observed in both patients with PD‐L1 CPS ≥ 5 and those with CPS < 1, with their mOS reaching 20.32 months and 17.64 months, respectively. In terms of safety, the most common grade 3 or higher TRAEs included neutropenia and other hematological abnormalities, with no new safety signals identified. This confirmed that the regimen achieves favorable efficacy and manageable safety in patients with G/GEJ adenocarcinoma across all PD‐L1 expression statuses [121].

ADCs have demonstrated significant efficacy in targeting HER2 in advanced GC, with initial results in the neoadjuvant stage showing improved pathological response [121]. ADCs targeting Claudin18.2, FGFR2 (such as TST001 and ZL‐121), combined with immunotherapy, are progressing rapidly in clinical trials [122, 123, 124, 125]. Multi‐target inhibitors (such as Regorafenib) inhibit tumor growth by blocking VEGFR/PDGFR pathways and may potentially synergize with immunotherapy in the future. Current research in advanced GC indicates improved survival rates [126]. Ongoing studies on other targets, such as FGFR2, cMET, TROP2, DKK1, and new targets, continue to progress [127]. Precise targeted therapy is becoming an inevitable direction for future drug research, promising more positive outcomes for widespread clinical application.

Tumor vaccines and CAR‐T cell therapy show promise as novel immunotherapy approaches in GC. Tumor vaccines activate specific T cells through personalized neoantigen selection (based on tumor mutation spectrum) and have been preliminarily verified for safety in lung cancer and other cancer types. However, their application in GC is limited by tumor heterogeneity and the challenge of selecting new antigen immunogenicity. CAR‐T therapy modifies T cells targeting HER‐2, Claudin18.2, and other antigens. Clinical trials in GC patients have shown tumor regression in some patients, but solid tumors still face challenges such as uneven target distribution, microenvironment suppression, and cytokine storms [128]. Future directions include improving the microenvironment through multi‐antigen target design and combination immunomodulators and exploring the synergistic value of immunotherapy with chemotherapy/radiotherapy in NAT to enhance the depth and persistence of immune therapy for GC.

### 3.4 Clinical Trials and Big Data Research

Multi‐center, large‐scale clinical trials will continue to drive the development of NAT. Rigorous trial design and multi‐center data validation will better evaluate the efficacy and safety of different treatment regimens. Additionally, big data analysis and artificial intelligence algorithms can uncover correlations between patient characteristics and treatment responses, supporting personalized treatment [129]. In the future, artificial intelligence will play a crucial role in predicting treatment plans and assessing efficacy.

## 4 Conclusion

With the rapid development of technology and the continuous deepening of research, precision and personalized therapy will become the mainstream trend. New drug development and optimization of combination therapy strategies are advancing together, with the emergence of multi‐target inhibitors, antibody‐drug conjugates, and novel immune checkpoint inhibitors. The strategic combination of different treatment methods and precise deployment will maximize efficacy and reduce toxicity. By performing fine molecular subtyping of GC using multi‐omics technologies, combined with liquid biopsy for real‐time, dynamic monitoring, it is expected that the most suitable treatment plan can be accurately selected, allowing each patient to benefit from personalized precision medicine. The increasing exchange between Eastern and Western medicine is expected to complement and merge, offering better choices for global GC patients. Medical workers, researchers, pharmaceutical companies, and policymakers worldwide need to collaborate to strengthen interdisciplinary efforts, integrate resources, and conduct large‐scale, multi‐center, prospective clinical trials and basic research. Through continuous exploration and innovation, optimizing treatment strategies, and overcoming existing challenges, it is expected that the prognosis of GC patients will significantly improve, writing a new chapter in the global fight against GC.

## Author Contributions

Jiaqi Zhou and Chengwang Guo made substantial contributions to the conception and design of the work. Jiaqi Zhou, Chengwang Guo, and Xiaojun Liu contributed to methodology development. Chengwang Guo, Jiaqi Zhou, and Peng Nie performed data curation, formal analysis, and investigation. Chengwang Guo, Jiaqi Zhou, and Mingze Zhang drafted the original manuscript. Peng Nie, Yuntao Ma, and Xiaojun Liu revised the manuscript critically. Yuntao Ma and Peng Nie secured funding. Jihong Wang supervised the study. All authors approved the final version and agreed to be accountable for all aspects of the work.

## Funding

This work was supported by the Major Project of the Gansu Provincial Science and Technology Program (Joint Research Fund) (Grant No. 24JRRA885), the Wu Jieping Medical Foundation Scientific Research Grant (Grant No. 320.6750.2025‐13‐59), and the Wuwei Clinical Research Center for Gastric Cancer (Grant No. 2023LC5011).

## Conflicts of Interest

The authors declare no conflicts of interest.

## Data Availability

No new original data were created for this study. All data used in the analysis are obtained from publicly accessible literature, with full citations included in the references.
